# Satisfaction with Health Care Interventions among Community Dwelling People with Cognitive Disorders and Their Informal Caregivers—A Systematic Review

**DOI:** 10.3390/healthcare8030240

**Published:** 2020-07-29

**Authors:** Agneta Malmgren Fänge, Björg Thordardottir, Metuge Ankhesnamon Ya-Nyonge, Connie Lethin

**Affiliations:** 1 Faculty of Medicine, Department of Health Sciences, Lund University, SE-221 00 Lund, Sweden; agneta.malmgren_fange@med.lu.se (A.M.F.); ankhesnamon@gmail.com (M.A.Y.-N.); connie.lethin@med.lu.se (C.L.); 2 Faculty of Health Sciences, Department of Occupational Therapy, Prosthetics and Orthotics, OsloMet—Oslo Metropolitan University, 0130 Oslo, Norway; 3 Faculty of Medicine, Department of Clinical Sciences, Clinical Memory Research Unit, Lund University, SE-221 00 Lund, Sweden

**Keywords:** cognitive impairment, informal caregivers, health care services, satisfaction

## Abstract

Informal caregivers have a leading role when implementing health care services for people with cognitive disorders living at home. This study aims to examine the current evidence for interventions with dual satisfaction with health care services for people with cognitive disorders and their caregivers. Original papers with quantitative and mixed method designs were extracted from two databases, covering years 2009–2018. Thirty-five original papers reported on satisfaction with health care services. The International Classification of Health Interventions (ICHI) was used to classify the interventions. Most interventions had a home-based approach (80%). Reduction in caregiver depression was the outcome measure with the highest level of satisfaction. Interventions to reduce depression or increase cognitive performance in persons with cognitive disorders gave the least satisfaction. Satisfaction of both caregivers and persons with cognitive disorders increased their use of services. In the ICHI, nearly 50% of the interventions were classified as activities and participation. A limited number of interventions have a positive effect on satisfaction of both the persons with cognitive disorders and the caregiver. It is important to focus on interventions that will benefit both simultaneously. More research is needed with a clear definition of satisfaction and the use of the ICHI guidelines.

## 1. Introduction

Mild cognitive disorders or impairments are characterized by a modest cognitive decline not fully interfering with independence in everyday life. However, additional effort and compensatory strategies on the part of the individual is required to perform activities of daily living (ADL’s) [1]. Major cognitive disorders, such as dementia, are characterized by a significant cognitive decline interfering with independence in ADL’s. Thus, over time, people with cognitive disorders (e.g., mild and major) increasingly need support and care in order to lead a good daily life at home. Informal caregivers are significantly important in this context and thus formal support is essential to reduce unmet needs and enhance satisfaction in this group of people [2].

Dissatisfaction with health care intervention may range from a desire to be listened to, to a desire for better communication in order to make the follow up processes more effective [3], while satisfaction with such an intervention may prevent early institutionalization and reduce health care costs for society [4]. Previous studies [5,6,7,8] have focused on expectations and experiences of people living with cognitive disorders and their caregivers at outpatient medical consultations, specifically reasons for expressed or unvoiced satisfaction or dissatisfaction with the interaction with the physician. A randomized controlled trial, involving 26 informal caregivers aimed at investigating the effects of cognitive behavioral therapy on psychological and physiological responses to stressful situations in caregiving. The results suggested a positive effect of the intervention on the general health of the caregivers, also resulting in better care [5]. A qualitative study involving data from the National Caregiver survey of 1269 United States (US) veterans with a dementia disease and their primary caregivers suggests that low caregiver satisfaction may indicate an impending breakdown in care recipients’ access to healthcare [6]. Another study on expectations, experiences, and tensions in a memory clinic involving in-depth post encounter interviews among 11 patients and 17 informal caregivers, found that patient expectations were opposing those of the caregivers [7]. Similarly, a qualitative study showed that people with mild cognitive impairments and their informal caregivers indicated differences in experiences of health care services, with the caregivers generally reporting more negative impressions of contact than the care recipients themselves [8].

To the best of our knowledge on current upgraded systematic reviews, none has so far focused on the satisfaction as an outcome of health care interventions among community living people with cognitive disorders and their informal caregivers. Since community-based health care interventions are based on local resources within municipalities, they tend to vary, both in form and service delivery. A common variant is support with ADL’s, prescription and implementation of assistive devices, blood pressure monitoring, offers to attend day care centers and other psychosocial intervention. More knowledge on the existing variance of interventions and user satisfaction can support health care professionals in identifying interventions that may enhance satisfaction among people with cognitive disorders and their informal caregivers. Furthermore, the International Classification of Health Interventions (ICHI) [9] may also support the identification and categorization of such interventions. ICHI is a derivative of the International Classification of Functioning, Disability and Health, ICF [10] and is being developed to provide a common tool for reporting and analyzing health interventions, mostly for research purposes but it can also be useful for guiding interventions in practice. ICHI covers all parts of the health system and contains a wide range of new material not found in national classifications. It defines intervention as an act performed for, with or on behalf of a person or a population with the purpose to assess, improve, maintain, promote or modify health, functioning or health conditions within four sections [9]:

- Interventions on Body Systems and Functions;
- Interventions on Activities and Participation Domains;
- Interventions on the Environment;
- Interventions on Health-Related Behaviors.

Providing interventions that result in dual satisfaction among people with cognitive disorders and their informal caregivers is a challenge. Thus, there is an increasing need for practical ways to improve satisfaction with health care interventions provided to community living people with cognitive disorders and their informal caregivers. Most of all, it is not known which interventions are perceived satisfactory and which are not.

Consequently, the aim of this study was to examine current research evidence on satisfaction with health care interventions among community living people with cognitive disorders and their informal caregivers. The following research questions guided this review:

1. What is the current evidence on satisfaction as an outcome of different health care interventions among community living people with cognitive disorders and their informal caregivers?
2. Which health care interventions are related to satisfaction among community living people with cognitive disorders and their informal caregivers?

## 2. Materials and Methods 

A detailed search in collaboration with an expert librarian was conducted using two databases, PubMed and the Cumulative Index to Nursing and Allied Health Literature (CINAHL), covering the years January 2009 to November 2018. The PICO [11] (P = participants, I = intervention, C = comparison, and O = outcome) format was used to develop and limit the search strategy as follows: P = community living people with cognitive disorders living at home and their informal caregivers (spouse, friends, family, close friend, siblings, partner or proxy); I = studies which evaluated satisfaction with health care interventions (psychosocial and physiological) among people with cognitive disorders and their informal caregivers; and C = not applicable; and O = outcomes in terms of satisfaction of the person with cognitive impairment or cognitive disease and the informal caregiver (e.g., acceptance, anxiety, attitudes, behavior, behavioral problems, burden, care and social support, caregiving role, confidence, cognitive performance, depression, experiences, feelings of belonging, frustration, functioning and dependency, memory, mood, perceived usefulness, quality of life or stress). Inclusion criteria were quantitative and mixed method studies written in the English language. Exclusion criteria were peer-reviewed primary studies that focused on the physical and medical effects of interventions without an outcome related to satisfaction and interventions among people living in nursing homes. Furthermore, study protocols, cross-sectional studies or studies that did not focus on intervention but rather on comparison of variables were excluded, as were commentaries, reviews, editorials, case studies, and papers with a qualitative design. In November 2018, a last search was made, this time limiting one of the search blocks to “Dementia” [MeSH] OR “Cognition Disorders [MeSH].

**Figure 1:**
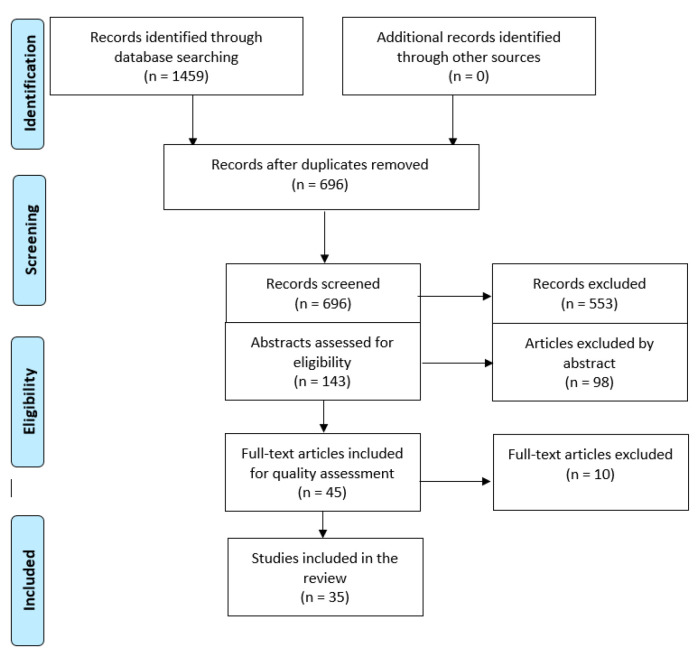
Flow diagram of the article selection process using the PRISMA flow diagram [12].

The search resulted in 224 articles from Pub Med and 492 articles in CINAHL (Figure 1). A total of 716 articles were transferred to EndNote manager and 21 were duplicates. Initially, AMF and MA independently screened the remaining 695 papers. Potentially eligible abstracts, (144) were retained and reviewed. Out of them, 98 did not reflect the aim of this review or the inclusion criteria and were thus excluded, resulting in 45 potential full-text papers. The full texts of the retained articles were then analyzed by all authors, who read them whilst strictly keeping the research questions in mind. In turn, the results were crossed checked by MA, CL and AMF until agreement was reached. In total, 35 papers were included in this review (Figure 1).

### Data Synthesis and Quality Assessment

The mixed methods appraisal tool (MMAT), revised version [13], was used to rate the quality of the included papers. MMAT applies to different quality criteria for different study designs, taking the unique characteristics of each design into consideration. Table 1 and Table 2 summarize data on the study design, context, participants, type of intervention, methods of data collection, and outcomes of interest in terms of satisfaction and quality of the included papers. Papers meeting 100% of the criteria were rated as top quality (5 stars *****); meeting 80% of the expected criteria were rated with four stars (****); meeting 60% of the criteria were rated with three stars (***); papers meeting 40 % of the criteria were rated with two stars (**); and finally papers meeting 20% of the criteria were rated with one star (*). AMF, CL and MA evaluated each paper separately and then compared the results. Disagreements between the authors (*n* = 9 papers) were discussed until consensus was reached. Lastly, interventions were categorized according to the ICHI [9].

## 3. Results

The 35 papers included a total of 3501 participants (Table 1), both caregivers and care recipients with a cognitive disorder. The papers described studies carried out in Asia [17,32,34], Europe [18,23,26,28,37,38,40,41,48], North America [16,19,20,21,22,24,25,27,29,30,31,35,36,39,42,43,44,45,46,47] and Australia [14,15,33]. In 28 studies, data were collected in the homes of the participants using a face-to-face or in-person approach, telephone sessions or both. Data were also collected in counselling rooms, online in one study or by a group approach. The group approach was used when activities such as aerobic exercise, meditation and meetings involved the use of machines and thus needed more space. In 17 studies, a randomized controlled design was applied, eleven studies used a quantitative non-randomized design, six a quantitative descriptive design and one paper described a mixed methods design study. For quality assessment, three papers were rated with one star (*); eight papers with two stars (**); twelve papers with three stars (***); nine papers with four stars (****); and three papers were of top quality (*****). See Table 1 for details.

Fifteen of the interventions in the included papers addressed only the caregiver. Nine interventions addressed persons with a cognitive disorder living alone, while 11 included caregiver and care recipient dyads. In over two thirds of the studies (24/35) the caregivers rated their satisfaction higher than before the interventions. In 10 of the 35 studies the caregivers experienced less depression [15,19,21,22,25,27,34,39,41,42], and seven studies reported that the caregivers were more satisfied with caregiver burden [14,17,19,21,22,31,34] than before the intervention. Six studies found reduction in caregiver anxiety or stress [15,16,25,27,36,39]. A reduction in caregiver burden was associated with lower levels of anxiety or depression in four studies [15,20,22,25].

Social support and use of formal services [20,24,35,45,47,48], better self-rated health [42,43], quality of life [25,32,34], satisfaction with caregiver role [15,17], regular meetings [28], satisfaction with the intervention [39] and increased confidence in managing difficult behavior of the care recipient [27] also resulted in increased satisfaction among caregivers.

The care recipients were satisfied with cognitive training [14], reduced behavioral problems [17], were less depressed and thus satisfied after home delivered psychosocial interventions [19,20,29,30] and counselling on communication [35]. Caregiver and care recipient satisfaction with and after the intervention were sometimes inconclusive. More caregivers than care recipients (2/3 vs. 1/3) were for example satisfied with the use of electronic tracking devices [37]. There were also different levels of satisfaction when the care recipient perceived that more attention was given to the caregiver [24]. Another study showed that while the intervention was reported beneficial for care recipient’s memory and ADL and reduced caregiver burden, the caregivers’ and care recipients’ mood did, however, not improve [14]. A reduction in caregiver depression was only related to less behavioral problems of care recipient [19]. Only one study found corresponding levels of satisfaction for both care recipient and caregiver, i.e., that caregiver’s experienced more independence and felt overall supported at the end of the intervention [35], which prompted continued use of services. Another two studies found that the interventions had positive effects for both the caregiver and the care recipient but to different extent [27,37]. In more than half of the studies, the presence of the caregivers during the interventions was necessary [15,16,17,20,24,26,27,29,31,32,35,37,40,41,43]. See Table 2 for details.

All interventions were classified according to ICHI (Table 2). Four interventions targeted body systems functions [14,44,45,47], 16 interventions targeted activities and participation [16,17,19,20,21,22,25,27,28,31,32,33,34,36,41,46], and 11 studies targeted interventions in the environment [15,18,23,24,26,35,37,38,42,47]. Four studies included interventions in more than one ICHI section [29,30,40,43]. In 16 of the studies [16,17,19,20,21,22,25,27,28,31,32,33,34,36,41,48], the interventions focused on learning new skills, applying knowledge, and self-care. These areas correspond to the domain of activities and participation according to the ICF [10]. Interventions contributing most towards satisfaction were those that were home-based [16,17,19,20,21,22,25,26,30,31], targeting activities and participation.

## 4. Discussion

Our findings indicate that interventions aimed at the population under study vary in terms of design, origin and outcomes targeted. Most of the interventions resulted in an enhanced satisfaction among both caregivers and care recipients. However, the results of the interventions in terms of satisfaction differed extensively between caregivers and care recipients, revealing the sometimes-complicated relationships that exist between them.

From a general perspective, key issues related to research in dementia are related to the difficulty to recruit people with dementia into studies. The tendency is thus to ignore the perspectives of people with dementia [49], instead the biomedical aspects of neuropathology and aspects of social interactions and contexts are put into focus [50,51,52,53]. In our study, most of the results focused the psychosocial aspect of care for people with dementia and their informal caregivers. One of our main findings is that the perspectives, worries and concerns of caregivers may affect the benefits and outcomes of interventions in the home, given that they are of capital importance in the care plan, even when they were not the target of intervention. It is, therefore, important to clearly distinguish between satisfaction of the caregiver and that of the care recipient when planning interventions, and to focus on interventions that will benefit both simultaneously. Since interventions in the home are becoming more common, also for people with cognitive disorders, the design of future studies can benefit from our findings. According to the study by Giese and Cote [54], consumer satisfaction is either an emotional or cognitive response to the product or experience of services. Satisfaction is a phenomenon coexisting with other consumption emotions and caregivers are of capital influence in the use of services offered to care recipients. For example, caregivers that are skeptical to support and services may hinder care recipients from fully using the services [33]. It is, therefore, relevant to gain caregiver confidence and participation [55]. Our findings are in line with Lopez et al. [56], which in their research on the effect of caregiver support interventions found that caregivers were important resources for community-dwelling frail elderly and need to be well supported.

Interestingly, home-based psychoeducational interventions that naturally targeted activities and participation as categorized by the ICHI [9] appeared to give greatest satisfaction to both caregiver and care recipient. Therefore, group support interventions should address both caregivers and care recipients while at the same time take into consideration the fact that their needs differ. An earlier study [57] showed for example that although caregivers found day care beneficial for their care recipients’ activity and participation, as well as for themselves, care recipients with behavioral problems and those who needed assistance with dressing and toileting are prone to discontinue day care, sometimes after only a few months’ attendance. More recently, Saks et al. [58] concluded that suitable community services may divert nursing home entry for certain individuals. Lethin et al. [59] also addressed the different context of care in exploring home care vs. nursing home care in rural vs urban settings. The study found that care recipients in home care have more behavioral problems than those in a nursing home. It also revealed that caregivers in urban areas report higher burden compared to those living in rural areas. The positive findings regarding the benefits of interventions focusing on caregivers are in line with the study by Lethin et al. [60] showing that caregivers that were satisfied with social services also experienced increased well-being over time. It is further supported that diminished caregiver well-being as well as their negative perception of quality of care predict increased burden [61]. These findings stress the need for an explicit focus on home-based interventions that benefit caregivers and care recipients.

### Strengths and Limitations

Although satisfaction is an important outcome of health care interventions, it is not so common in medical research [62]. This may be due to the complexity of the concept [12] and its measurability. In this context, it was not surprising that most studies included in our review applied no clear definition of satisfaction. Most importantly, in order to enhance outcome evaluation as well as comparison across studies satisfaction as a concept needs to be clearly defined by researchers before and after interventions are made.

This paper attempted to extract satisfaction with health care interventions as the main outcome measure. In an attempt to classify the interventions, the ICHI (9) was used. This classification is still under development but highly recommended by the WHO [9] as it may support global initiatives, such as the Sustainable Development Goals and Universal Health Coverage to provide information for patient safety and health system performance [9]. In this respect, this systematic review adds to current research by providing an example of how the ICHI can be applied.

A weakness of our study is the fact that our search strategy did not capture papers including studies conducted in Africa and South America; generalization of the results beyond the regions included is therefore difficult to make. Lepore et al. [49], in their systematic review also mentioned this limitation, highlighting the fact that people of African origin and other ethnic minority groups are under-represented in this kind of research. Moreover, the health care systems in different regions differ considerably in many aspects. Thus, our findings should be interpreted with caution. Moreover, since our study excluded people living in nursing homes, future studies should include the experiences of those people and their informal caregivers.

## 5. Conclusions

In summary, health care and social service interventions may have an adequate effect on the satisfaction of the caregiver and care recipient living at home. Most importantly, interventions that bring satisfaction to both parties may be beneficial in that it leads to continued use of health care and social services provided. Home-based psychoeducational interventions, targeting activities and participation, appears to result in the greatest satisfaction for both care recipient and caregiver. We thus can conclude that group support interventions should address both caregivers and care recipients, and to consider the fact that their needs differ. It is, therefore, important to distinguish between satisfaction of the care recipient and the caregiver when planning interventions, and to focus on interventions that will benefit both simultaneously. For research and practice purposes, the ICHI would harmonize the coding of interventions around the globe, in turn of added advantage to future intervention, planning and evaluation.

## Figures and Tables

**Table 1:** Description of study details, design and quality assessment.

Author, Year, Title, Country	ID	Study Design, Duration, MMAT Score, Participants (Number (*n*), Age)	Data Collection, Survey (Evaluation of) and Scales
Bahar-Fuchs et al. (2017) Tailored and Adaptive Computerized Cognitive Training in Older Adults at Risk for Dementia: A Randomized Controlled Trial. Australia	[14]	Randomized Controlled Trial, 8-12 weeks 2/*** Adults with mild cognitive impairment or mood related neuropsychiatric symptoms or both (*n* = 45, >65 years old)	Survey: computerized cognitive training tailored to individuals’ profile Scales: memory; awareness/meta-memory; mood and well-being; independence in day-to-day activities; and caregiver burden
Baker et al. (2012) Connecting through music: A study of a spousal caregiver-directed music intervention designed to prolong fulfilling relationships in couples where one person has dementia. Australia	[15]	Mixed methods, 6 weeks 4/* Couples where one partner had dementia (*n* = 10, 59–88 years old)	Survey: spousal caregiver-directed music intervention Scales: depression; anxiety; behavior; satisfaction with caregiver role; quality of the spousal relationship
Braddock et al. (2011) The effects of student home visits on activity engagement in persons with Alzheimer’s disease and related disorders. United States of America (USA)	[16]	Quantitative nonrandomized, 8 weeks 3/**** Person with dementia (*n* = 32, 69–92 years old)	Surveys: levels of constructive engagement, verbal utterances, and engagement in targeted activity Scales: cognition of the person with dementia; caregiver self-efficacy; and caregiver burden outside student visits; caregiver burden; and self-confidence
Cheung et al. (2015) Multicomponent intervention on enhancing dementia caregiver well-being and reducing behavioral problems among Hong Kong Chinese. China	[17]	Longitudinal experimental design, 12 sessions 3/**** Family dyads (*n* = 201, informal caregiver, 35–89 years old person with dementia, 56–97 years old)	Survey: caregivers taught to record the antecedents and consequences of behavioral problems and use of records to keep track of behavioral changes Scales: caregiver burden; depression; positive aspects of caregiving; occurrence and bother of behavioral problems
Chiatti et al. (2015) The UP-TECH project, an intervention to support caregivers of Alzheimer’s disease patients in Italy: preliminary findings on recruitment and caregiving burden in the baseline population. Italy	[18]	Quantitative descriptive, 12 months 4/***** Caregivers (*n* = 438, mean age 61.4 years)	Surveys: care and service use, informal caregiver burden and its determinants Scales: personal and instrumental activities of daily living (ADL); anxiety and depression; caregiver self-report of health status; and perceived social support
Czaja et al. (2018) Community REACH: An Implementation of an Evidence-Based Caregiver Program. USA	[19]	Longitudinal study, 6-months 3/*** Dyads (*n* = 146, caregiver > 65 years old, person with dementia <81 years old)	Survey: informal caregiver multicomponent psychosocial intervention program Scales: informal caregiver burden; depression; safety; use of social support; caregiving self- efficacy; positive aspects of caregiving; self-rated health; quality of life; and care recipients’ cognitive status; ADL; and observable behavioral problems
Czaja et al. (2013) A videophone Psychosocial Intervention for Dementia Care givers. USA	[20]	Randomized Controlled Trial, 5 months 2/*** Caregiver (*n* = 110, mean age 60.9 years)	Survey: technology based multi-component psychosocial intervention in-home and via videophone technology Scales: caregiver depression; burden; social support; perception of the caregiver experience; and care recipients’ cognitive status; and behavioral problems
Easom et al. (2013) A Rural Community Translation of a Dementia Caregiving Intervention. USA	[21]	Quantitative descriptive, 6 months 4/** Caregiver (*n* = 85, mean age 67 years)	Survey: impact of a multicomponent, evidence-based, tailored intervention for caregivers Scales: caregiver general questions; safety; health behavior; social support; stress; behavioral frustration; burden; depression; intervention evaluation; desire to institutionalize the care recipient; and program evaluation
Fortinsky et al. (2009) Dementia care consultation for family caregivers: Collaborative model linking an Alzheimer’s association chapter with primary care physicians. USA	[22]	Randomized Controlled Trial, 12 months 2/** Caregiver (*n* = 54/30, mean age 64.8/57.7 years)	Survey: efficacy of an individualized dementia care consultation Scales: caregiver burden; depression; physical health; satisfaction with intervention; and care recipients cognition; and problematic behavior
Frederiksen et al. (2014) Moderate-to-high intensity aerobic exercise in patients with mild to moderate Alzheimer’s disease. A pilot study. Denmark	[23]	Uncontrolled pre-post intervention test evaluation, 14 weeks 3/**** Care recipient (*n* = 9, mean age 71.9 years)	Surveys: physical exercise, care recipients maximum oxygen up take, expired gases, knee extension, chair stand test and experience of intensity and duration of the training program Scales: care recipients cognition; depression; ADL; quality of life; and quality of life of caregiver
Gaugler et al. (2011) The Memory Club: Providing Support to Persons with Early-Stage Dementia and Their Care Partners. USA	[24]	Uncontrolled pre-post-test evaluation, 10–13 weeks 3/**** Care recipient (*n* = 63, mean age 74.25 years) Caregiver (*n* = 61, mean age 69.16 years)	Survey: satisfaction of caregiver and care recipient Scales: caregiver effectiveness; self- rated stress; and care recipient anticipation of care; instrumental and personal ADL; and depressive symptoms
Jain et al. (2014) Feasibility of Central Meditation and Imagery Therapy for dementia caregivers. USA	[25]	Quantitative descriptive, 8 weeks 4/** Caregiver (*n* = 12, ≥45 years)	Survey: dementia caregivers reporting stress due to caregiving responsibilities Scales: caregiver depression; anxiety; quality of life enjoyment and satisfaction; insomnia; mindfulness; and credibility of therapy
Jansen et al. (2011) Effectiveness of case management among older adults with early symptoms of dementia and their primary informal caregivers. The Netherlands	[26]	Randomized Controlled Trial, 12 months 2/**** Dyads (*n* = 54/45, caregiver mean age 63.6/61.6; care recipient, mean age 82.1/81 years)	Survey: comparing case management and usual care Scales: caregiver’s sense of competence; quality of life; depression; burden; self-esteem; feelings; mastery; caregiver distress; and for the person with early signs of dementia cognition; quality of life; behavioral problems; ADL; social support; and self-care
Johnson et al. (2013) Treatment Outcomes of a Crisis Intervention Program for Dementia With Severe Psychiatric Complications: The Kansas Bridge Project. USA	[27]	Nonrandomized concurrent control outcome trial, 6 months 3/**** Dyads (*n* = 77/52, mean age 79.3/82.4 years)	Survey: community-integrated response to dementia crisis Scales: caregiver depression; behavioral problems; and support in ADL of the person with dementia; impact of the intervention on the person with dementia and caregivers; and dementia-related symptoms; and their impact on the current crisis
Johling et al. (2012) Does a Family Meetings Intervention Prevent Depression and Anxiety in Family Caregivers of Dementia Patients? The Netherlands	[28]	Randomized Controlled Trial, 1 year 2/**** Care recipient (*n* = 96/96, mean age 72.8/76.7 years) Caregiver (*n* = 96/96, mean age 67.8/71.1 years)	Survey: preventive effects of family meetings for primary caregivers Scales: caregiver depression or anxiety; burden; health-related quality of life; and for the person with dementia cognition; ADL; and behavioral symptoms
Kiosses et al. (2010) Home-Delivered Problem Adaptation Therapy (PATH) for Depressed, Cognitively Impaired, Disabled Elders: A Preliminary Study. USA	[29]	Randomized Controlled Trial, 12 weeks 2/*** Care recipient (*n* = 30, ≥65 years old)	Survey: home-delivered problem adaptation therapy versus home-delivered supportive therapy in reducing depression and disability Scales: cognition; executive dysfunction; comorbidity; anti-depressant medication; depression and disability; ADL; patient satisfaction with treatment
Kiosses et al. (2017) Negative Emotions and Suicidal Ideation during Psychosocial Treatments in Older Adults with Major Depression and Cognitive Impairment. USA	[30]	Randomized Controlled Trial, 12 weeks 2/*** Care recipient (*n* = 74, 65–95 years old)	Survey: relationship of negative emotions with suicidal ideation of problem adaptation therapy vs. supportive therapy Scales: depression; cognitive impairment; suicidal ideation
Kunik et al. (2017) Teaching Caregivers of Persons with dementia to address pain. USA	[31]	Randomized Controlled Trial, 12 months 2/*** persons with dementia and their caregivers (*n* = 203)	Surveys: the efficacy of preventing aggression with that of usual care in decreasing incidence of aggression and pain and improving depression, pleasant events, caregiver burden, and patient–caregiver relationship quality Scales: caregiver–patient relationship; depression; pain; pleasant activities; agitation; frequency and disruptiveness of behaviors for the person with dementia; caregiver burden; and satisfaction
Kuo et al. (2013) A home-based training program improves Taiwanese family caregivers’ quality of life and decreases their risk for depression. Taiwan	[32]	Randomized Controlled Trial, 6 months 2/*** Caregiver (*n* = 63/66, mean age 54.7/56.2 years)	Surveys: effects of a home-based caregiver training program on health-related quality of life and depressive symptoms for family caregivers of older persons with dementia Scales: caregiver health-related quality of life; and depression
Leach et al. (2015) Transcendental Meditation for the improvement of health and wellbeing in community-dwelling dementia caregivers [TRANSCENDENT]. Australia	[33]	Randomized Controlled Trial, 24 weeks 2/**** Caregiver (*n* = 8/9, mean age 69.4/63.2 years)	Surveys: improving psychological stress, quality of life, affect and cognitive performance with a transcendental meditation program Scales: health-related quality of life; stress; affect; adverse events; cost-effectiveness; and cognitive performance
Lee et al. (2012) Effects of home-based stress management training on primary caregivers of elderly people with dementia in South Korea. South Korea	[34]	Randomized Controlled Trial, 7 weeks 2/** Caregiver (*n* = 15/15, mean age 51.4/56.4 years)	Surveys: effect of stress management training on primary caregivers Scales: depression; stress; and life satisfaction
Lingler et al. (2016) Development of a Standardized Approach to Disclosing Amyloid Imaging Research Results in Mild Cognitive Impairment: Pilot testing. USA	[35]	Cross-sectional pre-post-test evaluation 3/** Dyads (*n* = 10, caregiver mean age 63.2 years; person with dementia mean age 78.6 years)	Survey: informational materials for use in pre-test counselling and post-test disclosures of amyloid imaging research Scales: health literacy
Llanque et al. (2015) The Family Series Workshop: A Community- Based Psycho-Educational Intervention. USA	[36]	Pre-post-test design, 6 weeks 3/***** Caregiver (*n* = 35, mean age 62.7 years)	Survey: evaluation of a community-based psychoeducational intervention Scales: coping; caregiving competence; and burden
Magnusson et al. (2014) Extended safety and support systems for people with dementia living at home: A descriptive study. Sweden	[37]	Cross-sectional pre-post-test, 8 months 4/***** Care recipient (*n* = 63, mean age 74.7 years) Caregiver (*n* = 62, mean age 62.2 years)	Survey: implementation of advanced electronic tracking, communication and emergency response technologies Scales: depression of the person with dementia and caregiver
McKechnie et al. (2014) The Effectiveness of an Internet Support Forum for Careers of People With Dementia. United Kingdom (UK)	[38]	Mixed method–pre-post cohort study, 12 weeks 5/*** Caregiver (*n* = 61, 22–86 years old)	Survey: impact of an online support Scales: anxiety; depression; and quality of relationship in caregiving
Paukert et al. (2010) Peaceful mind: an open trial of cognitive- behavioral therapy for anxiety in persons with dementia. USA	[39]	Open trial, 6 months 3/** Care recipient (*n* = 8, 67–89 years old) Caregiver (*n* = 8, no information of age)	Survey: feasibility and utility of the intervention and assessment procedures Scales: anxiety; depression; worry; satisfaction with treatment; and level of dementia and behavior problems of the person with dementia
Prick et al. (2015) The effects of a multi- component dyadic intervention on the psychological distress of family caregivers providing care to people with dementia: a randomized controlled trial. The Netherlands	[40]	Randomized Controlled Trial, 6-months 2/**** Dyads (*n* = 111, ≥ 55 years old)	Surveys: the effect of a multi-component intervention on caregivers’ mood, burden, general health, and salivary cortisol levels Scales: caregiver depression; mood; self-perceived pressure; self-rated general health; cognition and behavioral problems for the person with dementia
Schoenmarkers et al. (2010) Supporting Family Careers of Community-Dwelling Elder with Cognitive Decline. Belgium	[41]	Randomized controlled trial, 12 months 2/** Dyads (*n* = 32/27, caregiver, mean age 64.4/62.3 years)	Survey: a care counsellor, coordinating care during one year, will alleviate caregivers’ feelings of depression Scales: caregiver burden; and depression, neuropsychiatric symptoms; personal and instrumental ADL of the person with dementia
Simpson et al. (2010) Pilot Study of a Brief Behavioral Sleep Intervention for Caregivers of Individuals with Dementia: Pilot study. USA	[42]	Cross-sectional, 5 weeks 3/*** Caregiver (*n* = 10, mean age 63 years)	Survey: behavioral sleep intervention to improve caregiver sleep quality Scales: caregiver depression; health and sleep quality
Stanley et al. (2013) The Peaceful Mind Program: A Pilot Test of a Cognitive—Behavioral Therapy Based Intervention for Anxious Patients with Dementia. USA	[43]	Randomized Controlled Trial, 6 months 2/*** Dyads (*n* = 16/16, care recipient mean age 77.6/79.6 years)	Survey: a cognitive-behavioral therapy-based intervention for anxiety in dementia, relative to usual care Scales: caregiver anxiety; depression; quality of life and cognition; dementia rating; anxiety; quality of life; health and worry for the person with dementia
Steinberg et al. (2009) Evaluation of a home-based exercise program in the treatment of Alzheimer’s disease: The Maximizing Independence in Dementia (MIND) study. USA	[44]	Randomized Controlled Trial, 12 weeks 2/*** Care recipient (*n* = 14/13, mean age 76.5/74 years)	Survey: a home-based exercise intervention program to improve the functional performance of patients with Alzheimer’s disease Scales: functional performance; cognitive functioning; neuropsychiatric symptoms; caregiver burden; and quality of life
Steis et al. (2012) Detection of delirium in community-dwelling persons with dementia. USA	[45]	Randomized Controlled Trial, 3–45 days 2/** Care recipient (*n* = 13, mean age 76 years)	Survey: caregiver satisfaction with technology of electronically reported observations of delirium symptoms Scales: cognition; delirium symptoms; and dementia rating of the person with dementia
Sussman et al. (2009) The Influence of Community-Based Services on the Burden of Spouses Caring for their Partners with Dementia. Canada	[46]	Cross-sectional survey 4/* Caregiver (*n* = 46, mean age 76 years)	Survey: community services effect on the stress process for spousal caregivers Scales: caregiver burden; perceptions and use of community services; support from family and friends; experience of behavior disturbance; and independence in ADL of the person with dementia
Tappen et Hain (2014) The Effect of In-Home Cognitive Training on Functional Performance of Individuals with Mild Cognitive Impairment and Early-Stage Alzheimer’s Disease. USA	[47]	Randomized Controlled Trial, 12 weeks 2/* Dyads (*n* = 37/31, care recipient, mean age 80.9/81.8 years)	Survey: comparing in-home cognitive training program to life story interview Scales: caregiver depression; response on memory-related functional performance; language; caregiver mood, reaction and satisfaction; satisfaction with caregiving, and experience of behavior problems; and cognition and dementia rating of the person with dementia
Van Mierlo et al. (2012) Dementia coach: effect of telephone coaching on careers of community dwelling people with dementia. The Netherlands	[48]	Pre-post-experimental, 20 weeks 3/*** Caregiver (*n* = 21/25/8, mean age 63.5/62.3/69 years)	Survey: telephone coaching to support informal caregivers evaluated on burden and mental health Scales: caregiver burden; general health; severity of dementia; and neuropsychiatric symptoms

**Table 2:** Satisfaction with the interventions provided.

Author, Year	Type of Intervention	Domain of Intervention	Outcome	Satisfaction		Comments on Satisfaction	
				Informal Caregiver	Person with Cognitive Disorder	Informal Caregiver	Person withCognitive Disorder
Bahar-Fuchs et al., 2017 [14]	Computerized cognitive training program in persons with MrNPS and MCI	Cognition	Memory Mood	No	Yes No		Greater improvement of cognitive ability for both MrNPS and MCI
			Caregiver burden	Yes		Reduced caregiver burden for MrNPS only. Improved independence of ADL	
Baker et al., 2012 [15]	Music intervention to prolong fulfilling relationships in couples	Role functioning emotional	Anxiety	Yes		Reduced anxiety	
			Depression	Yes		Reduced depression	
			Behavior	Yes		Better spousal relationship	
			Satisfaction with care giving role	Yes		Increased caregiver satisfaction	
Braddock et al., 2011 [16]	Guided and targeted activities of everyday life directed by student and caregiver, based on persons’ needs past interests	Impact on the informal caregiver	Stress	Yes			
			Confidence	No			
Cheung et al., 2014 [17]	Intervention to enhance caregiver well-being and reduce behavioral problems in persons with cognitive disorders	Behavior and neuropsychiatric symptoms	Depression		Not totally	Reduction in depression only related to behavioral problems	
			Behavioral problems		Yes		
		Impact on the caregiver	Positive aspects of care	Yes		Reduction in all subscales of caregiver risks	
			Burden	Yes			
Chiatti et al., 2015 [18]	Care strategy to support community dwelling caregivers of persons with moderate Alzheimer’s Disease	Impact on the caregiver	Burden	No			
Czaja et al., 2018 [19]	An evidence based multipsychosocial intervention to provide information to caregivers on problem solving behavioral strategies	Impact on caregiver	Burden	Yes			Lower overall burden at 6 months and at follow up
			Depression	Yes			Less depression at 6 months and maintained at follow up
Czaja et al., 2013 [20]	Efficacy of technology based video psychosocial intervention among minority informal caregivers of persons with cognitive disorders	Impact on caregiver and person with cognitive disorders	Satisfaction with social support	Yes at 12 months	No at 6 months		Higher overall social support was observed at 12 months
			Use of formal care services	Yes			There was increased use of respite services at 12 months
Easom et al., 2013 [21]	Evidenced based educational support to caregivers of persons with cognitive disorders in a rural community for a risk free environment	Impact on caregiver	Burden	Yes			
			Frustration	Yes			Reduced after intervention
			Depression	Yes			
			Confidence	Yes			Increased as they learned new techniques
Fortinsky et al., 2009 [22]	A dementia care consultation intervention	Impact on caregiver	Depression	Yes			Significantly reduced
			Burden	Yes			Significantly reduced
			Satisfaction	Not totally			
Frederiksen et al., 2014 [23]	Balance training and aerobic exercise for persons with mild to moderate Alzheimer’s disease	Patient quality of life	Quality of life		No		
Gaugler et al., 2011 [24]	Therapeutic sessions to strengthen and improve dyad relationship and communication	Language and communication	Satisfaction with service	Yes	No	Caregiver satisfaction with memory club was high	
Jain et al., 2014 [25]	Meditation and guided imagery targeting dementia caregivers	Impact on caregiver	Quality of life	Yes			
			Anxiety	Yes		Anxiety decreased	
			Depression	Yes		Depression decreased	
Jansen et al., 2011 [26]	Case management intervention for patients with dementia and their caregivers	Impact on caregiver	Quality of life	No			
			Depressive symptoms	No			
			Burden		No		
		Patient quality of life	Caregivers satisfaction with older adult as a recipient	No			
			Feelings of belonging		No		
			Overall perception on quality of life		No		
Johnson et al., 2013 [27]	Interventions geared to support caregivers and persons with cognitive disorder with neuro-psychiatric symptoms to help prevent re- hospitalization	Behavior and neuropsychiatric symptoms	Patient anxiety		Yes		
			Patient depression		Yes		
			Caregiver anxiety	Yes			
		Impact on caregiver	Comorbidities (depression)	Yes			
			Confidence in ability to manage difficult behavior	Yes			
Johling et al., 2012 [28]	Psychoeducational family meetings with caregivers for problem solving	Impact on caregiver	Anxiety	No			
			Comorbidities (depression)	No			
			Burden	No			
			Quality of life	No			
			Satisfaction with meetings	Yes		Satisfaction with the meetings among caregivers was high.	
Kiosses et al., 2010 [29]	Home delivered problem adaptation therapy for depressed cognitive disabled elders	Behavioral and neuropsychiatric symptoms	Depression		Yes		
		Functioning and dependency of patient	Client satisfaction		Yes		
Kiosses et al., 2017 [30]	Home delivered psychosocial intervention to reduce suicidal ideation and improve negative emotions in older adults with cognitive impairment	Behavior and neuropsychiatric symptoms	Depression		Yes		
			Anxiety		Yes		
		Patient quality of life	Satisfaction with treatment		Yes		
Kunik et al., 2017 [31]	Home psychosocial and educational intervention to evaluate pain and enhance communication	Informal caregiver	Depression	No	No		
			Burden	Yes			
			Satisfaction and perceptions of usefulness				
Kuo et al., 2013 [32]	Training program to reduce care giver quality of life and reduce depression in caregivers	Patient quality of life	Health related quality of life	Yes			
		Behavior and neuropsychiatric symptoms	Depressive symptoms	Yes			
		Impact on the caregiver	Quality of life	Yes			
Leach et al., 2015 [33]	Transcendental meditation to improve health and well- being in community dwelling caregivers	Impact on the caregiver	Psychological stress	No			
			Quality of life	No			
			Cognitive performance	No			
Lee et al., 2012 [34]	Home based stress management training for caregivers of persons with dementia to reduce physical and psychological vulnerability	Impact on the caregiver	Burden	Yes			
			Depression	Yes			
			Life satisfaction	Yes			
Lingler et al., 2016 [35]	Pre-test counselling and post-test disclosure of amyloid brain research results in PwMCI	Language and communication	Satisfaction with the service	Yes	Yes		
Llanque et al., 2015 [36]	A psycho educational intervention to avoid preinstitutionalization in PwADRD	Impact on caregiver	Stress	Yes			
Magnusson et al., 2014 [37]	Caregivers, persons with cognitive disorder, professional careers perspectives of use of electronic tracking device on personal integrity	Social issues	Attitudes towards health care services	Positive			
			Views about usability	⅔ were satisfied	⅓ were satisfied	Caregivers were more satisfied than users	
McKechnie et al., 2014 [38]	An internet forum to share information and get advice for persons with cognitive disorder and their caregivers	Impact on caregiver	Stress	No			
			Anxiety	No			
			Experiences	Both positive and negative			
Paukert et al., 2010 [39]	Cognitive behavioral therapy, providing calming and breathing skills to reduce anxiety in caregivers	Behavioral and neuropsychiatric symptoms	Anxiety/sleep patterns	Yes			
			Depression	Yes			
			Satisfaction with the intervention	Yes			
Prick et al., 2015 [40]	A psycho educational communication intervention providing physical training, support and pleasant activities for persons with cognitive disorder	Behavioral and neuropsychiatric symptoms	Caregiver distress	No			
Schoenmarkers et al., 2010 [41]	Home care to relieve depression in caregivers of persons with cognitive disorder	Career quality of life	Depression	Yes			
Simpson et al., 2010 [42]	Cognitive behavioral sleep intervention	Behavioral and neuropsychiatric symptoms	Depression	Yes		Reduced	
		Career quality of life	Self-rated health	Yes		Satisfaction could be due to over rating.	
Stanley et al., 2013 [43]	Cognitive behavioral therapy for anxiety, breathing and sleeping skills for person with dementia and caregivers	Behavioral and neuropsychiatric symptoms	Anxiety		Yes at 3 months No at 6 months		
			Depression		No		
		Impact on career	Self-rated health	Yes at 3 months No at 6 months			
		Quality of life	Client satisfaction		Yes		
Steinberg et al., 2009 [44]	Home based exercise program for persons with dementia	Behavioral and neuropsychiatric symptoms	Depression		No		
Steis et al., 2012 [45]	Using smart phones in detecting delirium in persons with dementia	Impact on informal caregiver	Satisfaction with service	Yes			
Sussman et al., 2009 [46]	Influence of community services on burden of spouses	Impact on informal caregiver	Burden	No			
Tappen and Hain, 2014 [47]	Cognitive training for PwMCI	Impact on informal caregiver	Perceived satisfaction	Yes			
Van Mierlo et al., 2012 [48]	Telephone coaching to reduce burden and mental health problems of caregivers of CDPwD	Impact on caregiver	Satisfaction with services	Yes			

Abbreviations: ADL: activities in daily living, MCI: mild cognitive impairment, MrNPS: mood related neuropsychiatric Symptoms, CDPwD: community dwelling people with dementia, PwMCI: persons with mild cognitive impairment, PwADRD: persons with Alzheimer’s disease and related dementias.

## References

[1] American Psychiatric Association (2013). Diagnostic and Statistical Manual of Mental Disorders (DSM-5VR).

[2] Park M., Choi S., Lee S.J., Kim S.H., Kim J., Go Y., Lee D.Y. (2018). The roles of unmet needs and formal support in the caregiving satisfaction and caregiving burden of family caregivers for persons with dementia. Int. Psychogeriatr..

[3] Karnieli-Miller O., Werner P., Neufeld-Kroszynski G., Eidelman S. (2012). Are you talking to me?! An exploration of the triadic physician–patient-companion communication within memory clinic encounters. Patient Educ. Counc..

[4] Leifer B.P. (2003). Diagnosis of Alzheimer’s Disease: Clinical and Economic Benefits. J. Am. Geriatr. Soc..

[5] Aboulafia-Brakha T., Suchecki D., Gouveia-Paulino F., Nitrini R., Ptak R. (2014). Cognitive-behavioural group therapy improves psychophysiological marker of stress in caregivers of patients with Alzheimer disease. Aging Ment. Health.

[6] Thorpe J.M., van Houtven C.H., Sleath B.L. (2009). Barriers to outpatient care in community-dwellling elderly with dementia: The role of caregiver life satisfaction. J. Appl. Gerontol..

[7] Karnieli-Miller O., Werner P., Aharon-Peretz J., Sinoff G., Eidelman S. (2012). Expectations, experiences and tensions in the memory clinic: The process of diagnosis disclosure of dementia within a triad. Int. Psychogeriatr..

[8] Dean K., Jenkinson C., Wilcock G., Walker Z. (2014). Exploring the experiences of people with mild cognitive impairment and their caregivers with particular reference to healthcare—A qualitative study. Int. Psychogeriatr..

[9] World Health Organization (2019). ICHI The New Interventions Classification for Every Health System. International Classification of Health Interventions.

[10] World Health Organization International Classification of Functioning, Disability and Health. https://www.who.int/classifications/icf/en/.

[11] Boudin F., Nie J.Y., Bartlett J.C., Grad R., Pluye P., Dawes M. (2010). Combining classifiers for robust PICO element detection. BMC Med. Inform. Decis. Mak..

[12] Moher D., Liberati A., Tetzlff J., Altman D.G. (2009). The PRISMA Group. Preferred reporting items for systematic reviews and meta-analyses: The PRISMA statement. PLoS Med..

[13] Hong Q.N., Pluye P., Fàbregues S., Bartlett G., Boardman F., Cargo M., Dagenais P., Gagnon M.-P., Griffiths F., Nicolau B. (2018). The mixed methods appraisal tool (MMAT) version 2018 for information professionals and researchers. Educ. Inform..

[14] Bahar-Fuchs A., Webb S., Bartsch L., Rebok G., Cherbuin N., Anstey K.J. (2017). Tailored and adaptive computerized cognitive training in older adults at risk for dementia: A randomized controlled trial. J. Alzheimers Dis..

[15] Baker F., Grocke D., Pachana N. (2012). Connecting through music: A study of a spousal caregiver—Directed music intervention designed to prolong fulfilling relationships in couples where one person has dementia. AJMT.

[16] Braddock B., Phipps E. (2011). The effects of student home visits on activity engagement in persons with Alzheimer’s disease and related disorders. Am. J. Recreat. Ther..

[17] Cheung K.S., Lau B.H., Wong P.W., Leung A.Y., Lou V.W., Chan G.M., Schulz R. (2015). Multicomponent intervention on enhancing dementia caregiver well-being and reducing behavioral problems among Hong Kong Chinese: A translational study based on REACH II. Int. J. Geriatr. Psychiatry.

[18] Chiatti C., Rimland J.M., Bonfranceschi F., Masera F., Bustacchini S., Cassetta L., Lattanzio F., UP-TECH research group (2015). The UP-TECH project, an intervention to support caregivers of Alzheimer’s disease patients in Italy: Preliminary findings on recruitment and caregiving burden in the baseline population. Aging Ment. Health.

[19] Czaja S.J., Lee C.C., Perdomo D., Loewenstein D., Bravo M., Moxley J.H., Schulz R. (2018). Community REACH: An implementation of an evidence-based caregiver program. Gerontologist.

[20] Czaja S.J., Loewenstein D., Schulz R., Nair S.N., Perdomo D. (2013). A videophone psychosocial intervention for dementia caregivers. Am. J. Geriatr. Psychiatry.

[21] Easom L.R., Alston G., Coleman R. (2013). A rural community translation of a dementia caregiving intervention. Online J. Rural. Nurs. Health Care.

[22] Fortinsky R.H., Kulldorff M., Kleppinger A., Kenyon-Pesce L. (2009). Dementia care consultation for family caregivers: Collaborative model linking an Alzheimer’s association chapter with primary care physicians. Aging Ment. Health.

[23] Frederiksen K.S., Sobol N., Beyer N., Hasselbalch S., Waldemar G. (2014). Moderate-to-high intensity aerobic exercise in patients with mild to moderate Alzheimer’s disease: A pilot study. Int. J. Geriatr. Psychiatry.

[24] Gaugler J.E., Gallagher-Winker K., Kehrberg K., Lunde A.M., Marsolek C.M., Ringham K., Thompson G., Barclay M. (2011). The Memory Club: Providing support to persons with early-stage dementia and their care partners. Am. J. Alzheimers Dis. Other Demen..

[25] Jain F.A., Nazarian N., Lavretsky H. (2014). Feasibility of central meditation and imagery therapy for dementia caregivers. Int. J. Geriatr. Psychiatry.

[26] Jansen A.P., van Hout H.P., Nijpels G., Rijmen F., Dröes R.M., Pot A.M., Schellevis F.G., Stalman W.A., van Marwijk H.W. (2011). Effectiveness of case management among older adults with early symptoms of dementia and their primary informal caregivers: A randomized clinical trial. Int. J. Nurs. Stud..

[27] Johnson D.K., Niedens M., Wilson J.R., Swartzendruber L., Yeager A., Jones K. (2013). Treatment outcomes of a crisis intervention program for dementia with severe psychiatric complications: The Kansas Bridge Project. Gerontologist.

[28] Joling K.J., van Marwijk H.W., Smit F., van der Horst H.E., Scheltens P., van de Ven P.M., Mittelman M.S., van Hout H.P. (2012). Does a family meetings intervention prevent depression and anxiety in family caregivers of dementia patients? A randomized trial. PLoS ONE.

[29] Kiosses D.N., Arean P.A., Teri L., Alexopoulos G.S. (2010). Home-delivered problem adaptation therapy (PATH) for depressed, cognitively impaired, disabled elders: A preliminary study. Am. J. Geriatr. Psychiatry.

[30] Kiosses D.N., Gross J.J., Banerjee S., Duberstein P.R., Putrino D., Alexopoulos G.S. (2017). Negative emotions and suicidal ideation during psychosocial treatments in older adults with major depression and cognitive impairment. Am. J. Geriatr. Psychiatry.

[31] Kunik M.E., Snow A.L., Wilson N., Amspoker A.B., Sansgiry S., Morgan R.O., Ying J., Hersch G., Stanley M.A. (2017). Teaching Caregivers of Persons with Dementia to Address Pain. Am. J. Geriatr. Psychiatry.

[32] Kuo L.M., Huang H.L., Huang H.L., Liang J., Chiu Y.C., Chen S.T., Kwok Y.T., Hsu W.C., Shyu Y.I. (2013). A home-based training program improves Taiwanese family caregivers’ quality of life and decreases their risk for depression: A randomized controlled trial. Int. J. Geriatr. Psychiatry.

[33] Leach M.J., Francis A., Ziaian T. (2015). Transcendental Meditation for the improvement of health and wellbeing in community-dwelling dementia caregivers [TRANSCENDENT]: A randomised wait-list controlled trial. BMC Complement Altern. Med..

[34] Lee Y.-R., Sung K., Kim Y.-E. (2012). Effects of home-based stress management training on primary caregivers of elderly people with dementia in South Korea. Dementia.

[35] Lingler J.H., Butters M.A., Gentry A.L., Hu L., Hunsaker A.E., Klunk W.E., Mattos M.K., Parker L.S., Roberts J.S., Schulz R. (2016). Development of a standardized approach to disclosing amyloid imaging research results in mild cognitive impairment. J. Alzheimers Dis..

[36] Llanque S.M., Enriquez M., Cheng A.L., Doty L., Brotto M.A., Kelly P.J., Niedens M., Caserta M.S., Savage L.M. (2015). The family series workshop: A community-based psychoeducational intervention. Am. J. Alzheimers Dis. Other Demen..

[37] Magnusson L., Sandman L., Rosén K.G., Hanson E. (2014). Extended safety and support systems for people with dementia living at home. J. Assist. Technol..

[38] McKechnie V., Barker C., Stott J. (2014). The effectiveness of an Internet support forum for carers of people with dementia: A pre-post cohort study. J. Med. Internet Res..

[39] Paukert A.L., Calleo J., Kraus-Schuman C., Snow L., Wilson N., Petersen N.J., Kunik M.E., Stanley M.A. (2010). Peaceful Mind: An open trial of cognitive-behavioral therapy for anxiety in persons with dementia. Int. Psychogeriatr..

[40] Prick A.E., de Lange J., Twisk J., Pot A.M. (2015). The effects of a multi-component dyadic intervention on the psychological distress of family caregivers providing care to people with dementia: A randomized controlled trial. Int. Psychogeriatr..

[41] Schoenmakers B., Buntinx F., Delepeleire J. (2010). Supporting family carers of community-dwelling elder with cognitive decline: A randomized controlled trial. Int. J. Fam. Med..

[42] Simpson C., Carter P.A. (2010). Pilot study of a brief behavioral sleep intervention for caregivers of individuals with dementia. Res. Gerontol. Nurs..

[43] Stanley M.A., Calleo J., Bush A.L., Wilson N., Snow A.L., Kraus-Schuman C., Paukert A.L., Petersen N.J., Brenes G.A., Schulz P.E. (2013). The peaceful mind program: A pilot test of a cognitive-behavioral therapy-based intervention for anxious patients with dementia. Am. J. Geriatr. Psychiatry.

[44] Steinberg M., Leoutsakos J.M., Podewils L.J., Lyketsos C.G. (2009). Evaluation of a home-based exercise program in the treatment of Alzheimer’s disease: The Maximizing Independence in Dementia (MIND) study. Int. J. Geriatr. Psychiatry.

[45] Steis M.R., Prabhu V.V., Kolanowski A., Kang Y., Bowles K.H., Fick D., Evans L. (2012). Detection of delirium in community-dwelling persons with dementia. Online J. Nurs. Inform..

[46] Sussman T., Regehr C. (2009). The influence of community-based services on the burden of spouses caring for their partners with dementia. Health Soc. Work.

[47] Tappen R.M., Hain D. (2014). The effect of in-home cognitive training on functional performance of individuals with mild cognitive impairment and early-stage Alzheimer’s disease. Res. Gerontol. Nurs..

[48] Van Mierlo L.D., Meiland F.J., Dröes R.M. (2012). Dementelcoach: Effect of telephone coaching on carers of community-dwelling people with dementia. Int. Psychogeriatr..

[49] Lepore M., Shuman S.B., Wiener J.M., Gould E. (2017). Challenges in Involving People with Dementia as Study Participants in Research on Care and Services.

[50] Lloyd V., Gatherer A., Kalsy S. (2006). Conducting qualitative interview research with people with expressive language difficulties. Qual. Health Res..

[51] O’Connor D., Phinney A., Smith A., Small J., Purves B., Perry J., Drance E., Donnelly M., Chaudhury H., Beattie L. (2007). Personhood in dementia care. Developing a research agenda for broadening the vision. Dementia.

[52] Smebye K.L., Kirkevold M., Engedal K. (2012). How do persons with dementia participate in decision making related to health and daily care? A multi-case study. BMC Health Serv. Res..

[53] Wilkinson H., Wilkinson H. (2002). Including people with dementia in research: Methods and motivations. The Perspectives of People with Dementia: Research Methods and Motivations.

[54] Giese J.L., Cote J.A. (2000). Defining consumer satisfaction. Acad. Mark. Sci. Rev..

[55] Goeman D., Renehan E., Koch S. (2016). What is the effectiveness of the support worker role for people with dementia and their carers? A systematic review. BMC Health Serv. Res..

[56] Lopez-Hartmann M., Wens J., Verhoeven V., Remmen R. (2012). The effect of caregiver support interventions for informal caregivers of community-dwelling frail elderly: A systematic review. Int. J. Integr. Care.

[57] Måvall L., Malmberg B. (2007). Day care for persons with dementia: An alterantive for whom?. Dementia.

[58] Saks K., Tiit E.-M., Verbeek H., Raamat K., Armolik A., Leibur J., Meyer G., Zabalegui A., Leino-Kilpi H., Karlsson S. (2015). RightTimePlaceCare Consortium. Most appropriate placement for people with dementia: Individual experts’ vs. expert groups’ decisions in eight European countries. J. Adv. Nurs..

[59] Lethin C., Hallberg I.R., Vingare E.-L., Giertz L. (2019). Persons with Dementia Living at Home or in Nursing Homes in Nine Swedish Urban or Rural Municipalities. Healthcare.

[60] Lethin C., Renom-Guiteras A., Zwakhalen S., Soto-Martin M., Saks K., Zabalegui A., Challis D.J., Nilsson C., Karlsson S. (2017). Psychological well-being over time among informal caregivers caring for persons with dementia living at home. Aging Ment. Health.

[61] Lethin C., Leino-Kilpi H., Bleijlevens M., Stephan A., Martin M.S., Nilsson K., Nilsson C., Zabalegui A., Karlsson S. (2020). Predicting caregiver burden in informal caregivers caring for persons with dementia living at home—A follow-up cohort study. Dementia.

[62] Mira J.J., Aranaz J. (2000). Patient satisfaction as an outcome measure in health care. Med. Clin..

